# Discovery and Validation of Serum MicroRNAs as Early Diagnostic Biomarkers for Prostate Cancer in Chinese Population

**DOI:** 10.1155/2019/9306803

**Published:** 2019-08-25

**Authors:** Ji Lyu, Lin Zhao, Fubo Wang, Jin Ji, Zhi Cao, Huan Xu, Xiaolei Shi, Yasheng Zhu, Chao Zhang, Fei Guo, Bo Yang, Yinghao Sun

**Affiliations:** ^1^Department of Urology, Shanghai Changhai Hospital, Naval Medical University (Second Military Medical University), China; ^2^Division of Urology and Transplantation, Department of Surgery, Sichuan Academy of Medical Sciences and Sichuan Provincial People's Hospital and Hospital of The University of Electronic Science and Technology of China, Chengdu, Sichuan 610072, China

## Abstract

Prostate cancer (PCa) incidence has been rising in Chinese population. Current PSA-based biopsy has limited positive rate. Our research focused on development of serum markers for the diagnosis of PCa in patients with elevated PSA. miRNAs are found to be aberrantly expressed in many types of cancer. They are readily detectable in plasma and serum. Currently, miRNAs are being evaluated as potential prognostic and diagnostic tools for many types of cancer. We first profiled global serum miRNAs in a pilot set of PCa and benign prostatic hyperplasia (BPH) cases undergoing TRUS-guided prostate biopsy due to elevated PSA levels. A total of 20 differentially expressed miRNAs were discovered by high throughput microarray for further testing using qRT-PCR. In the training phase with 78 PCa and 77 BPH cases, miR-365a-3p, miR-4286, miR-424-5p, miR-27a-3p, and miR-29b-3p were found to have potential diagnostic value. The Logistics regression equation was established by 5 parameters including PSA, prostate volume, miR-4286, miR-27a-3p, and miR-29b-3p and ROC analysis of this model was made with AUC up to 0.892 (95% CI: 0.832-0.937, sensitivity 78.95%, and specificity 92.21%). The panel had excellent diagnostic performance and its significance was confirmed in 100 serum samples in the validation cohort. Overall, we found a panel of serum microRNAs that have considerable clinical significance in detecting early-stage prostate cancer. When combined with PSA and prostate volume, these microRNAs exhibit favorable diagnostic potency.

## 1. Introduction

Prostate cancer (PCa) is among the most frequently diagnosed and lethal cancer type in men of western countries [1]. On the contrary, the incidence of PCa in Asian men is much lower. However, the incidence and mortality rates of PCa have been increasing rapidly in China, as well as other Asian countries [2]. In the past few decades, prostate specific antigen (PSA), a protein secreted by prostate cells, has been adopted as prostate cancer biomarker worldwide. PSA has a relatively good sensitivity; however, its specificity is low due to the fact that many benign conditions can also cause elevated PSA levels. Its low specificity has raised the argument that routine use of PSA has resulted in overdiagnosis, overtreatment, and increased health care cost without lowering the PCa mortality rate significantly [3, 4]. It is reported that, in patients from western countries with PSA of 4-10ng/ml, the positive biopsy rate is around 20% [5]. And the positive biopsy rate in Chinese patients with PSA of 4-10ng/ml is about 26% [6]. Thus, it is warranted to look for more specific biomarkers for patients with elevated PSA levels.

MicroRNAs (miRNAs) are a group of small endogenous RNA molecules with a length of 19-23 nucleotides that have no coding capacities for proteins [7]. They were predicted to regulate up to 30% of the protein-coding genes [8]. They play pivotal functions in lots of biological processes, including proliferation, differentiation, apoptosis, and so on. They are also found to be aberrantly expressed in many types of cancer. They can act as both oncogenes and tumor suppressors by regulating cell cycle, DNA damage response, cellular senescence, and apoptosis [9]. Currently, miRNAs are being evaluated as potential prognostic and diagnostic tools for prostate and many other types of cancer [10].

To date, few publications investigated the diagnostic potential of miRNAs in prostate cancer. In this study, we aimed to compare the serum microRNA level profile of prostate cancer patients' and BPH patients' sera using qRT-PCR and find out differentially expressed microRNAs to help improve current diagnostic potency.

## 2. Materials and Methods

### 2.1. Patients and Control Subjects

**Figure 1:**
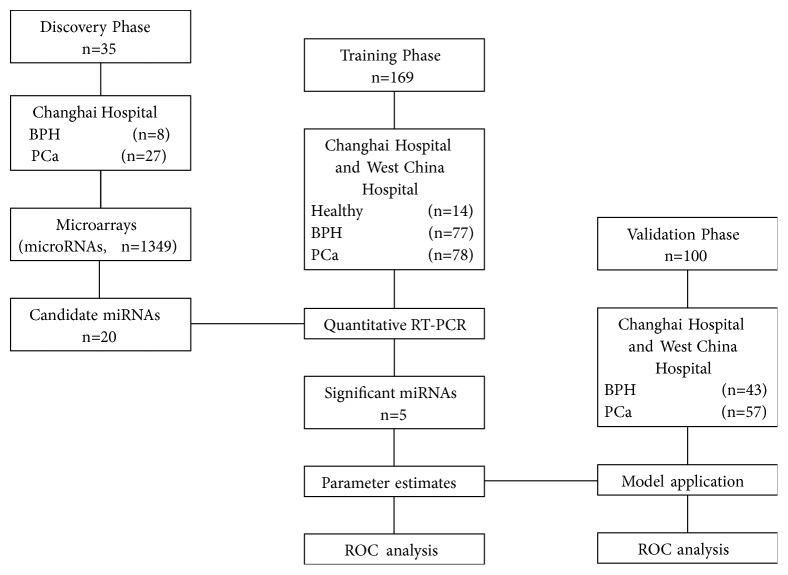
Flow chart of study design. BPH, benign prostatic hyperplasia; PCa, prostate cancer.

All human studies were carried out according to protocols approved by the Institutional Review Board (IRB) at Shanghai Changhai Hospital and West China Hospital. Written consent forms were obtained from all participants involved in the study. All patients included were not treated with antiandrogen therapy or 5*α*-reductase inhibitor before surgery. In the discovery phase, we included 27 patients with clinically localized PCa who underwent radical prostatectomy and 8 patients with benign prostate hyperplasia (BPH) who underwent transurethral resection of the prostate. In the training phase, we included 78 PCa patients, 77 BPH patients and 14 healthy volunteers. In the validation phase, 57 PCa and 43 BPH patients were included (Figure 1).

### 2.2. Serum Preparation

In general, around 6ml of venous blood was collected from each patient. All samples were left at room temperature for 30 mins to clot. Then serum was separated from the whole blood within 2h by centrifugation at 1600g for 10 min. Only the upper 2/3 serum was collected. Samples with hemolysis were discarded. Serum was stored at -80°C until subsequent analysis.

### 2.3. RNA Isolation

Serum total RNA from PCa patients, BPH patients and normal controls was isolated using mirVana PARIS Kit (Ambion 1556, USA) following manufacturer's protocol. 350*μ*l serum was used for each sample. NanoDrop ND-2000c (Thermo Fisher Scientific, Inc., Wilmington, DE) was adopted to measure the purities and concentrations of isolated RNAs. Extracted RNAs with OD 260/280 between 1.8 and 2.1 were adopted for subsequent analysis. Samples with more than 500ng were excluded, as there could be potential contamination from white blood cells.

### 2.4. MicroRNA Microarray Expression Profiling

Total RNA (100 ng) from the pooled samples of patients with PCa(27) and BPH(8) were used for microRNA microarray profiling. Total RNA was labeled by means of the miRNA Complete Labeling and Hyb Kit (Affymetrix) and hybridized on the Human MicroRNA Microarray Kit (release 16.0), which contains 60,000 probes for 1,205 human and 144 human viral miRNAs. Hybridization signals were detected by means of the Affymetrix Microarray Scanner, and the scanned images were analyzed with the use of Affymetrix Feature Extraction Software [11].

### 2.5. Quantitative RT-PCR

Taqman microRNA assays (Applied Biosystems) were used to quantify miRNA expression as previously described [12]. We used the Taqman MicroRNA Reverse Transcription Kit according to the manufacturer's instructions. miR-1228-3p is used as internal control [13]. The Reverse Transcription (RT) reactions are performed using a single miRNA-specific stem-loop RT-primer. As we need a lot of cDNA for qRT-PCR, the total reaction volume is doubled. 50ng of total RNA is combined in the 30*μ*l (total reaction volume) RT reaction with 0.30*μ*l 100×dNTP mix, 2*μ*l MultiScribe RT enzyme, 3*μ*l 10× RT-PCR buffer, 0.38*μ*l RNase-inhibitor, and 6*μ*l 5× specific RT-primer (the remainder of the reaction is made up of RNA and nuclease-free water). The RT reactions were incubated on a T100™ Thermal Cycler (Bio-Rad): the reaction mixture was incubated at 16°C for 30 minutes, followed by an incubation step at 42°C for 30 minutes, inactivated at 85°C for 5 minutes, then held at 4°C. Abundant miRNAs can be detected and accurately quantitated with reduced total RNA input. The quantitative PCR was run on ABI StepOne Plus(Applied Biosystems) using a two-step PCR protocol with an initial denaturation step at 95°C for 10 minutes, followed by 40 cycles with a denaturation step at 95°C for 2 minutes, and an annealing/extension step at 60°C for 60 seconds. The qRT-PCR data analysis was performed in StepOne Software v2.1 (Applied Biosystems, California, USA).

### 2.6. Statistical Analysis

Independent-sample t test is used for analyzing age. For comparison of miRNA ratios, PSA, f/tPSA, and prostate volume of different patient groups, we used the nonparametric unpaired Mann-Whitney-U test. The diagnostic power of every possible miRNA ratio combination (Target A/Target B = 2^−ΔCt^) was analyzed by receiver operating characteristic (ROC) curves. For all statistical tests, two-sided P values ≤ 0.05 were considered as statistically significant. In order to find all differentially expressed microRNAs, two-sided P values ≤ 0.1 were considered as statistically significant in the training phase. Statistical analyses were performed using SPSS statistical software (Version 19.0, USA).

## 3. Results

### 3.1. Discovery of Differentially Expressed MicroRNAs

We analyzed 1,205 human miRNAs and 144 viral miRNAs between BPH and PCa group using Human miRNA Microarray Kit Release 16.0, 8x60K. Among the serum samples, 8 are from BPH patients and 27 are from PCa patients (patient characteristics are presented in Table 1). Using Mann-Whitney U Test, a miRNA is considered as differentially expressed when P<0.05, FC>2.0 and miR-1228-3p/100>1. A total of 20 candidate human miRNAs are identified for further testing via qRT-PCR, including

**Figure 2:**
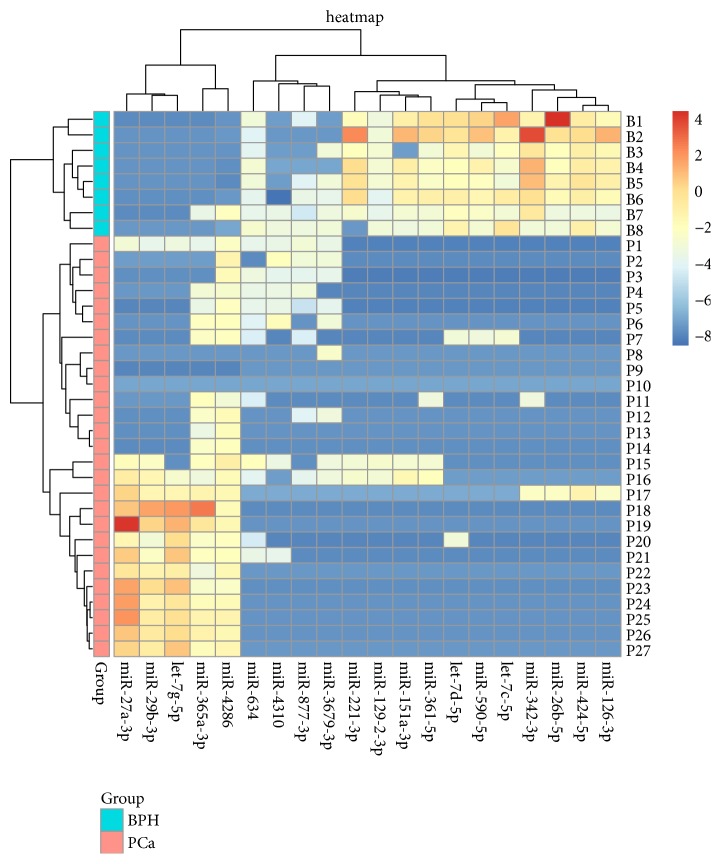
Heatmap showing differential miRNA expression in PCa blood samples (left lower: pink) compared with BPH blood samples (left upper: blue) from our pilot study.

miR-129-2-3p, miR-361-5p, miR-634, miR-590-5p, miR-342-3p, let-7d-5p, let-7g-5p, miR-26b-5p, miR-365a-3p, miR-4286, miR-424-5p, let-7c-5p, miR-29b-3p, miR-877-3p, miR-126-3p, miR-151a-3p, miR-221-3p, miR-3679-3p, miR-4310, and miR-27a-3p, as is shown in the heatmap (Figure 2).

### 3.2. Evaluation of the Stable Expression of the Screened miRNAs

Initially, we would like to see whether all 20 miRNAs could be detected by qRT-PCR in serum samples. We tested in 46 serum samples, including 19 from PCa patients, 13 from BPH patients, and 14 from normal control. Among them, miR-126-3p, miR-151a-3p, miR-221-3p, miR-3679-3p, miR-4310, and miR-634's ct value is more than 40 and miR-129-2-3p's ct value is between 36 and 40. These 7 miRNAs were excluded for further evaluations.

### 3.3. Individual Evaluation of Discovered miRNAs' Differential Expression between Groups

Up till now, 19 PCa, 13 BPH, and 14 normal serum samples have been used. We included another 23 PCa and 10 BPH serum samples to compare the differential expression of these miRNAs between groups using Mann-Whitney U test. Of the discovered 20 miRNAs, 7 miRNAs are excluded due to difficulty detecting. 13 miRNAs were tested in these samples. In order not to miss some significant miRNAs due to small sample size, we used P value less than 0.1 as statistically significant in this step. Between PCa and BPH, miR-365a-3p, miR-4286, let-7c-5p, and miR-29b-3p are differentially expressed (P<0.1). Between PCa and normal, miR-365a-3p, let-7c-5p, and miR-27a-3p are differentially expressed (P<0.1). Between BPH and normal, miR-4286 and miR-877-3p are differentially expressed (P<0.1) (Table 2). Finally, miR-365a-3p, miR-4286, miR-424-5p, let-7c-5p, miR-29b-3p, miR-877-3p, and miR-27a-3p are used for additional analysis.

### 3.4. Expression Profile of the Discovered miRNAs in Patients with Elevated PSA

**Figure 3:**
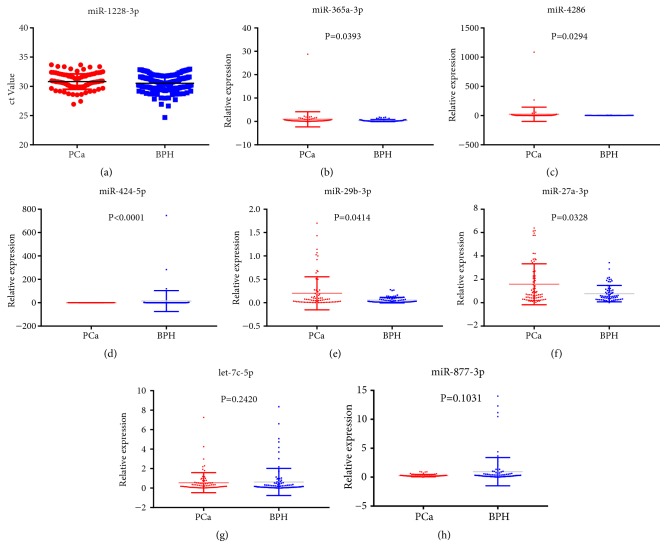
The ct value of internal control (miR-1228-3p, (a)) and relative expression of 7 miRNAs including miR-365a-3p (b), miR-4286 (c), miR-424-5p (d), miR-29b-3p (e), miR-27a-3p (f), let-7c-5p (g), and miR-877-3p (h) in PCa and BPH patients.

**Figure 4:**
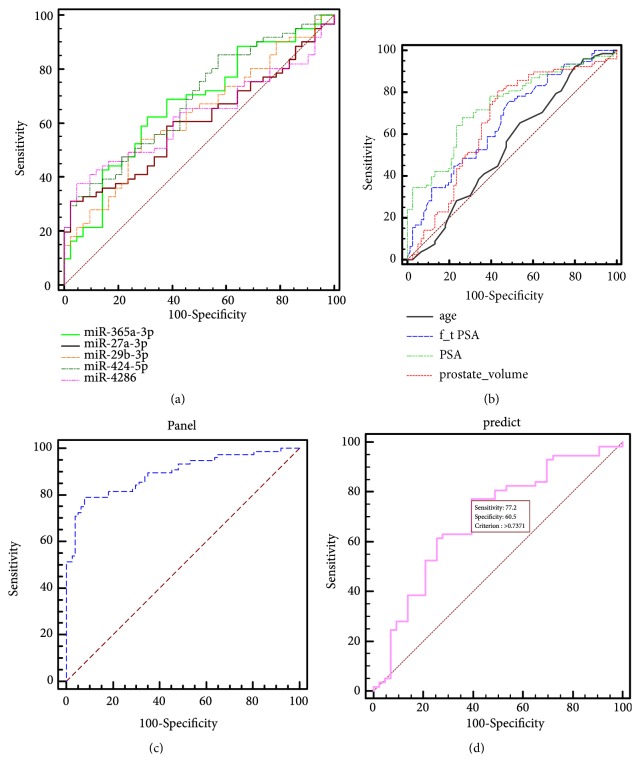
ROC analysis of different parameters. (a) ROC analysis of miRNAs including miR-365a-3p, miR-4286, miR-424-5p, miR-29b-3p, and miR-27a-3p; (b) ROC analysis of PSA, f/t PSA, age, and prostate volume; (c) ROC analysis using the microRNA panel (miR-4286, miR-27a-3p, and miR-29b-3p) combined with clinical parameters (PSA and prostate volume); (d) ROC analysis using the Logistics regression equation in the validation cohort.

Another set of 90 patients with elevated PSA were used for analysis. Among them, 36 are PCa patients and 54 BPH patients. miR-1228-3p was used as internal control. miR-1228-3p is stably expressed in PCa and BPH patients' serum samples, with no statistical difference between the two groups(P>0.05, Figure 3(a)). Among the 7 miRNAs, miR-365a-3p (P=0.0393); miR-4286 (P=0.0294); miR-424-5p (P<0.0001); miR-29b-3p (P=0.0414) and miR-27a-3p (P=0.0328) are statistically different between the two groups, while let-7c-5p (P=0.2420) and miR-877-3p (P=0.1031) are not(Figures 3(b)–3(h)). The diagnostic accuracy of these five differentially expressed miRNAs was measured by ROC analysis. Among them, miR-424-5p has the highest AUC,0.671 (95% CI: 0.590-0.746) and miR-29b-3p has the lowest AUC, 0.593 (95% CI: 0.513-0.669) (Figures 4(a)). miR-4286 has the highest sensitivity (67.5%) and miR-27a-3p has the highest specificity (97.18%) (Table 3).

### 3.5. Evaluation of Diagnostic Performance of Clinical Variables in the Training Dataset

We combined the abovementioned samples, including 78 PCa samples and 77 BPH samples. 14 normal samples were excluded as our target patients are those with elevated PSA levels. Four clinical variables were included in our study, namely age, PSA, f/tPSA and prostate volume. The AUC for age, PSA, f/tPSA, and prostate volume are 0.546, 0.738, 0.668, and 0.657, respectively (Figure 4(b), Table 4).

### 3.6. Development of the Predictive miRNA Panel

In the previous analysis, clinical variables including age, PSA, f/tPSA and prostate volume, and 5 miRNAs including miR-365a-3p, miR-4286, miR-424-5p, miR-29b-3p, and miR-27a-3p are statistically different between PCa and BPH patients. Therefore, a stepwise logistic regression model to estimate the risk of being diagnosed with PCa was established combining clinical variables and our miRNAs. In our model, PSA, prostate volume, miR-4286, miR-27a-3p, and miR-29b-3p turned out to be significant predictors of PCa (Table 5). The predicted probability of being diagnosed with PCa from the logit model based on the 5 parameters, logit (P=PCa) = -2.614 + 0.096 x PSA - 0.034 x (prostate volume) + 0.341 x miR-4286 + 0.875 x miR-27a-3p + 5.218 x miR-29b-3p was used to construct the ROC curve. The diagnostic performance for the established miRNA panel was evaluated by ROC analysis. The AUC for the microRNA panel was 0.892 (95% CI, 0.832 to 0.937) with a sensitivity of 78.95% and specificity of 92.21% (Figure 4(c)).

### 3.7. Application of the Predictive miRNA Panel to an Independent Validation Cohort of 100 Patients

The miRNA panel we established was used to predict the diagnostic value for PCa in an independent cohort of 100 patients (55 samples are from West China Hospital and 45 from Shanghai Changhai Hospital) for validation. Among the serum samples, 57 are from PCa patients and 43 from BPH patients. ROC analysis was used to evaluate the miRNA panel's diagnostic performance. The AUC for the microRNA panel was 0.70 (95% CI, 0.60 to 0.78) with a sensitivity of 77.19% and specificity of 60.47% (Figure 4(d)). The panel's positive predictive value is 69%, while its negative predictive value is 66%.

## 4. Discussion

In China, the incidence of prostate cancer has been on the steady rise over the past decades. Unlike western countries, many of the diagnosed cases in China are in advanced stage, making the mortality/incidence rate of Chinese PCa patients more than 40%. PSA is a widely adopted screening tool in western countries. It has gained popularity in China in recent years. In the United States, the mortality rate for prostate cancer has dropped by 51% from 1993 to 2016 due to early diagnosis and treatment [14]. Then the PCa mortality stabilized during 2013 through 2016 [15]. However, PSA has a low specificity to PCa and PSA-based biopsy has relatively low positive rate, making many people undergo unnecessary biopsy. The TRUS-guided biopsy is an invasive procedure and would make people suffer. In this study, we aimed to identify potential diagnostic biomarkers in the circulation that are noninvasive and easy to use and evaluate their diagnostic value in PCa.

miRNAs are widely involved in tumor biology, including tumor initiation, development, drug resistance, metastasis and diagnosis. It has been reported that miRNAs are differentially expressed among cancerous, adjacent noncancerous and normal tissues [16]. Mitchell PS et al. reported that miRNAs are readily detectable in blood and in a remarkably stable form that will not be degraded by endogenous RNase activity [17]. It has been widely reported that miRNAs in circulation (plasma, serum or exosomes) could be biomarkers for PCa diagnosis, progression and prognosis [18–20]. High throughput microarray of miRNAs is the first step to detect tumor specific miRNAs. Rather than using a single miRNA, a panel combining several miRNAs has been used for disease diagnosis. Zhou J et al. used a panel of miRNAs including miR-122, miR-192, miR-21, miR-223, miR-26a, miR-27a and miR-801 to diagnose hepatic cell carcinoma at early stage. The microRNA panel was able to differentiate HCC from healthy (AUC=0.941), chronic hepatitis B (AUC=0.842), and cirrhosis (AUC=0.884), respectively. Zheng G et al. used a panel including miR-19a-3p, miR-223-3p, miR-92a-3p and miR-422a in colorectal cancer early diagnosis and Lian F et al. used a panel including miR-195-5p, miR-199a-3p, miR-320a, and miR-374a-5p as noninvasive biomarker for osteosarcoma diagnosis [21, 22]. They all achieved AUC more than 0.8.

In our study, we first demonstrated that most of the discovered miRNAs could be easily detected in serum using qRT-PCR. miR-1228-3p, the internal control we used, is a well-recognized internal control among many studies. The expression levels of our discovered miRNAs are compared to that of miR-1228-3p to be normalized. In the training phase, we found that miR-365a-3p, miR-4286, miR-424-5p, miR-29b-3p, and miR-27a-3p are statistically different between PCa and BPH groups. Their AUC are similar to that of PSA. Recently, Fumihiko Urabe et al. published a microarray analysis of serum miRNAs addressing the miRNAs profile of Japanese PCa patients. By analyzing their dataset, we found that 4 of the 5 differentially expressed miRNAs, namely miR-365a-3p, miR-4286, miR-424-5p and miR29b-3p, are also differentially expressed in their cohort, supporting these miRNAs' significance (Supplementary [Supplementary-material supplementary-material-1]) [23]. Among the 5 differentially expressed miRNAs, miR-424-5p, miR-29b-3p, and miR-27a-3p have a relatively good specificity. Our final logistic model included PSA, prostate volume, miR-4286, miR-27a-3p and miR-29b-3p. The panel has an AUC of 0.892 in the training phase and 0.7 in the multicenter validation phase. The difference in the AUC in-between may be due to a relatively small sample size in the validation phase and the high PSA level in patients from West China hospital which may cause biases. Larger sample cohort may demonstrate better diagnostic potency of our panel.

The three miRNAs we included in our final model have been found to be frequently deregulated among various cancer types. miR-4286 is found to be able to greatly upregulated in melanoma and metastatic cutaneous squamous cell carcinoma formalin-fixed paraffin-embedded tissue samples, suggesting its tumor promoting function [24, 25]. miR-27a-3p was reported to be frequently overexpressed in gastric cancer tissues and cell lines. It functions as an oncogene and promotes gastric cancer progression both in vitro and in vivo through downregulation of BTG2, demonstrating its ability to modulate malignant biological behavior [26]. miR-27a-3p was also found to be overexpressed in peripheral blood mononuclear cells (PBMCs) of pancreatic cancer patients. Combination of PBMC miR-27a-3p with serum CA 19-9 achieved an AUC of 0.886 with a sensitivity of 85.3% and specificity of 81.6% for the diagnosis of pancreatic cancer [27]. Paola Ulivi et al. found that circulating basal levels of miR-29b-3p was significantly associated with progression-free survival and overall survival in patients with metastatic colorectal cancer treated with Bevacizumab [28]. All the above reports suggest that these serum miRNAs might be released from cancer cell. Thus, these miRNAs have potential to be biomarkers for cancer.

Our discovered miRNAs are quite different from a previous report [29]. In their report, let-7e, let-7c, and miR-30c were found to be downregulated and miR-622 and miR-1285 upregulated in PCa patients. A number of factors could contribute to this difference. First, their study used plasma sample while our study used serum sample. It was reported that miRNAs may be released from blood cells into serum during coagulation process, resulting possible variations between plasma and serum miRNAs [30]. Second, different platform was adopted for discovery. They used Illumina's Human v2 miRNA microarray and we used Human miRNA Microarray Kit Release 16.0 from Agilent. Differential microRNA expression has been reported among different microarray platforms [31, 32]. However, miRNA levels in circulation depend on factors more than tumor type alone. They are also affected by chronic diseases, inflammation, and so on. Therefore, a discrepancy of miRNAs may exist.

There are also some limitations in our study: All patients included in our cohort are Asians, while Caucasians, hispanics, and African American's serum samples were not included. Our discovered miRNAs in serum is also quite different from a previously report, which mainly included western country patients [33]. It has been reported that racial differences of microRNAs exist in many types of diseases, such as hypertension, colon cancer, triple-negative breast cancer, endometrial cancer, and so on [34–37]. Therefore, these microRNAs need to be further evaluated in people other than Asians to see their diagnostic potency. The sample size we used to establish the panel was enough; however, larger sample size in future may better optimize the parameters. Furthermore, the prognostic value of this panel needs to be elucidated in future larger independent cohort.

In conclusion, our study demonstrates the use of a panel of serum miRNAs as noninvasive biomarkers for differentiating PCa from BPH in Chinese patients with elevated PSA.

## Figures and Tables

**Table 1:** Demographic characteristics of patients with BPH and PCa.

Clinical Index		PCa	BPH
Sample size		27	8
Age	mean, year	68.7	65.2
	median, year	70.0	65.5
PSA	mean, ng/ml	23.12	15.66
	median, ng/ml	9.66	11.81
Gleason Score (cases)	≤6	16	
	7	11	
	≥8	5	

**Table 2:** Expression profiles of candidate microRNAs on qRT-PCR in serum samples.

microRNA	PCa VS. BPH	PCa VS. Nor	BPH VS. Nor
miR-365a-3p	0.0239	0.0963	0.8812
miR-4286	0.0222	0.8545	0.0092
miR-590-5p	0.3248	0.9437	0.5679
miR-342-3p	0.6984	0.5684	0.5878
miR-361-5p	0.2538	0.3792	0.7727
miR-424-5p	<0.001	0.4443	0.0031
let-7d-5p	0.5951	0.4938	0.1978
let-7c-5p	0.0913	0.0509	0.7437
let-7g-5p	0.938	0.4856	0.4285
miR-26b-5p	0.9556	0.5166	0.4967
miR-29b-3p	0.0901	0.5166	0.4967
miR-877-3p	0.1274	0.1137	0.035
miR-27a-3p	0.1584	0.0716	0.3286

PCa: prostate cancer; BPH: benign prostate hyperplasia; Nor: normal; VS.: versus.

**Table 3:** ROC analysis of 5 miRNAs.

miRNA	AUC	Sensitivity (%)	Specificity (%)	95% CI	Cut-off value^*∗*^
miR-365a-3p	0.600	45.80	80.90	0.514-0.676	0.2062
miR-4286	0.610	67.50	52.78	0.527-0.688	3.7230
miR-424-5p	0.671	31.94	94.87	0.590-0.746	0.0135
miR-29b-3p	0.593	26.92	95.24	0.513-0.669	0.1415
miR-27a-3p	0.601	28.21	97.18	0.518-0.681	2.1588

*∗* Cut-off value: the value was relative expression level of targeted miRNA compared with expression level of miR-1228-3p.

**Table 4:** ROC analysis of clinical variables.

Clinical Index	AUC	Sensitivity (%)	Specificity (%)	95% CI	Cut-off Value
AGE (year)	0.546	92.31	19.48	0.464-0.626	58
PSA (ng/ml)	0.738	67.95	74.03	0.661-0.805	11.38
f/t	0.668	75.64	52.00	0.587-0.742	0.17
Prostate Volume (ml)	0.657	80.77	55.84	0.577-0.731	42.68

**Table 5:** Logistic regression parameter estimates.

Parameter	Coefficient	Std. error	P	OR^*∗*^	95% CI^&^	
					Lower Bound	Upper Bound
Constant	-2.614					
PSA	0.096	0.025	0.001	1.100	1.047	1.156
Prostate volume	-0.034	0.012	0.004	0.967	0.944	0.9895
miR-4286	0.341	0.105	0.001	1.406	1.146	1.727
miR-29b-3p	5.218	2.334	0.025	184.558	1.904	17888.198
miR-27a-3p	0.875	0.275	0.001	2.399	1.399	4.116

*∗* OR: odds ratio; & CI: confidence intervals.

## Data Availability

The data used to support the findings of this study are available from the corresponding author upon request.

## Abbreviations

PCa: Prostate cancer
BPH: Benign prostatic hyperplasia
ROC: Receiver operating characteristic
AUC: Area under curve.

## Supplementary Materials

Supplementary Materials Supplementary Figure 1: after analyzing Fumihiko Urabe's dataset in a Japanese cohort, available through the Gene Expression Omnibus database (GSE112264)), we found that our results are quite similar. In both studies, miR-365a-3p (P<0.0001), miR-4286 (P=0.0002), miR-424-5p (P=0.0004), and miR29b-3p (P=0.0061) are differentially expressed while let-7c-5p (P=0.1730) is not.
