# Supplementary figures and images for: Discovery and Validation of Serum MicroRNAs as Early Diagnostic Biomarkers for Prostate Cancer in Chinese Population
Source: Biomed Res Int. 2019 Aug 25;2019:9306803. doi: 10.1155/2019/9306803 (PMC6732591; doi:10.1155/2019/9306803)

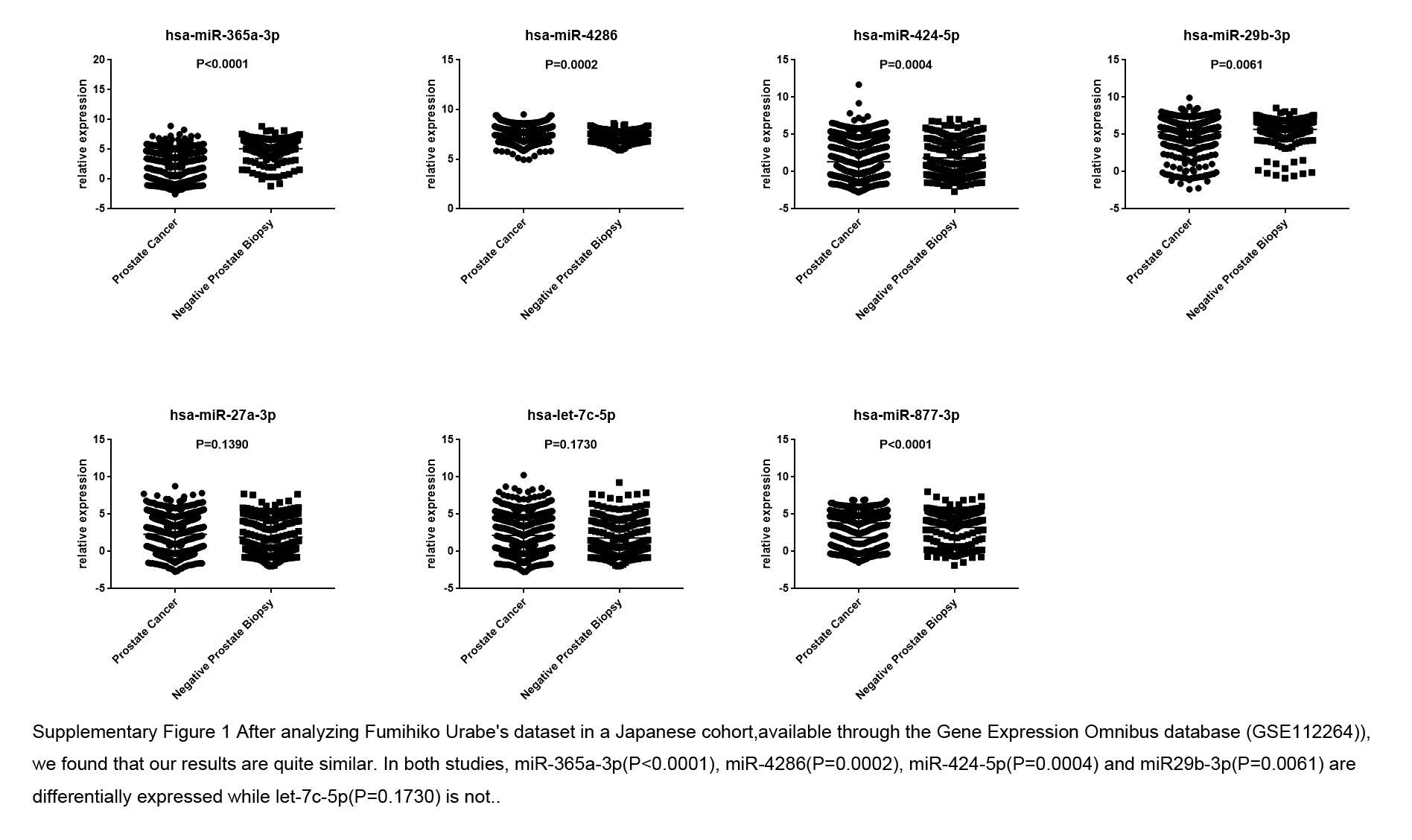

Supplement: Supplementary Materials — Supplementary Figure 1: after analyzing Fumihiko Urabe's dataset in a Japanese cohort, available through the Gene Expression Omnibus database (GSE112264)), we found that our results are quite similar. In both studies, miR-365a-3p (P<0.0001), miR-4286 (P=0.0002), miR-424-5p (P=0.0004), and miR29b-3p (P=0.0061) are differentially expressed while let-7c-5p (P=0.1730) is not. [file 9306803.f1.jpg]
